# Feasibility Study of Manufacturing Hydraulic Fittings Using Additive Manufacturing Technologies: Comparative Analysis of FDM and SLA Methods

**DOI:** 10.3390/ma19040799

**Published:** 2026-02-18

**Authors:** Jakub Backiel, Pawel Dzienis, Karol Golak, Przemysław Zamojski, Maciej Rećko, Rafał Grądzki, José Emiliano Martínez, Rogelio Valdés

**Affiliations:** 1 Faculty of Mechanical Engineering, Bialystok University of Technology, Wiejska 45C, 15-351 Białystok, Polandk.golak@pb.edu.pl (K.G.); p.zamojski@pb.edu.pl (P.Z.); m.recko@pb.edu.pl (M.R.); r.gradzki@pb.edu.pl (R.G.); 2 Department of Engineering Studies for Innovation, Universidad Iberoamericana Ciudad de México, Prol. Paseo de la Reforma 880, CDMX 01219, Mexico; jose.martinez@ibero.mx (J.E.M.); jose.valdes@ibero.mx (R.V.)

**Keywords:** additive manufacturing, 3D printing, FDM, SLA, hydraulic fittings, leak testing, PETG, photopolymer resin, dimensional accuracy, leak-tightness

## Abstract

This paper investigates the feasibility of manufacturing hydraulic fittings using additive manufacturing (AM) technologies, specifically Fused Deposition Modeling (FDM) and Stereolithography (SLA). The study addresses the environmental challenge of material waste in conventional fitting production by exploring 3D printing as an alternative manufacturing method. Hydraulic fittings were designed using CAD software: SolidWorks 2022 and fabricated using FDM with PETG (Polyethene Terephthalate Glycol) material and SLA with UV-sensitive photopolymer resin. In present studies, on-destructive leak testing was conducted in accordance with PN-EN 1254-4 and PN-EN 1254, at pressures ranging from 0.1 to 1.0 bar. Dimensional accuracy analysis revealed shrinkage of approximately 1% for SLA-printed parts and 2% for FDM-printed parts. Microscopic examination at 50× and 80× magnification showed superior thread quality in SLA samples compared to FDM, which exhibited visible layer separation and material porosity. Leak testing demonstrated that while the brass reference fitting maintained complete seal integrity, both 3D-printed variants failed to achieve leak tightness under operational pressures, with structural failure occurring at 1.0 bar during tightening. The study showed that FDM with PETG material and SLA with UV-sensitive photopolymer resin, despite achieving acceptable dimensional tolerances (±1–2%), do not meet hydraulic leak tightness requirements at pressures exceeding 0.5 bar in their raw state after printing. The results suggest that alternative material formulations (e.g., carbon fiber-reinforced PEEK for FDM or epoxy engineering resins for SLA) warrant further investigation. Potential avenues for improvement include advanced surface treatment, optimization of printing parameters, and modifications to thread geometry to reduce interthread gaps.

## 1. Introduction

Three-dimensional printing has been used since the 1980s and has since gained popularity. Since around 2009, 3D printers have become accessible to a broader range of users, not just large companies specializing in the production of mechanical system components. The largest producer of 3D printers is the United States of America [1]. Three-dimensional printing is an additive manufacturing technique. There are many 3D printing methods, such as FDM, SLS, and SLA. It involves gradually depositing molten layers of material, one atop another, based on a previously created virtual model of the object. One advantage of 3D printing is the ability to use recycled materials, which significantly increases material circulation and, consequently, reduces production costs [2]. Recent studies have demonstrated that PETG retains mechanical properties across multiple recycling cycles, with only minor degradation after six rounds of reprocessing [3]. Three-dimensional printing also adheres to technical standards, ensuring high-quality, precisely manufactured elements [4,5]. To operate a 3D printer, one must first learn to use computer-aided design (CAD) software. This is essential for correctly programming the path that the tool will follow [6]. The next step is to develop a basic understanding of physics and the mechanical properties of materials to select the optimal material for the application’s specific requirements.

Additive manufacturing has evolved significantly in recent years, with increasing interest in the production of functional parts beyond prototyping. The technology offers design flexibility, waste reduction, and the ability to create complex geometries that are difficult or impossible to achieve with traditional manufacturing methods. However, challenges remain in achieving the dimensional accuracy, surface quality, and mechanical properties required for demanding applications such as hydraulic systems [7,8].

FDM (Fused Deposition Modeling) is a process that uses thermoplastic filaments to produce parts. The print bed for this method should be heated to prevent the material from shrinking too quickly. In an FDM printer, the bed usually moves along the *Z*-axis (up and down), while the print nozzle moves along the *X* and *Y* axes [9]. There are also printers with work surfaces that can be adjusted to different angles (relative to the X and Y axes), making it easier to print complex parts. The thickness of the applied layer depends on the diameter of the nozzle through which the material is deposited. Layer thickness affects the precision and quality of the print. The most commonly used materials for the FDM method are PETG, PET, PLA, and ABS. When creating parts with complex shapes, it is necessary to add supports, which can be challenging to remove. Typical dimensional tolerances for FDM printing range from ±0.5% to ±0.5 mm, depending on part geometry and material properties [10].

The removal or minimization of supports is possible with the SLA method [11,12]. This method also enables relatively high part quality and geometric accuracy [9,13]. SLA technology achieves tolerances of approximately ±0.5% (±0.2 mm), making it one of the more accurate additive manufacturing methods [10]. To achieve optimal component performance, it is essential to set parameters such as layer thickness, curing speed, and temperature [14]. In the SLA method, the part is printed on a worktable submerged in a tank containing photopolymer resin. A laser beam or concentrated UV light cures the material layer by layer, forming the part. The material layer height depends on the step size of the worktable’s movement in the chamber. The table moves along the *Z*-axis, while the laser or UV light system moves along the X and Y axes. After the printing process is complete, the part is further cured with UV light. Due to its properties and the variety of available resins, SLA printing is most commonly used in medicine [15]. Recent research indicates that SLA parts can achieve superior leak tightness relative to FDM for specific geometries, though performance depends on surface integrity and post-processing.

Hydraulic fittings are among the most important components in fluid-transfer systems. The most critical aspect of hydraulic systems is maintaining their sealing integrity, which is primarily influenced by the quality of threaded connections. These systems often operate under high pressure, making it challenging to ensure a high level of sealing. There are technical standards that specify how leakage tests on pipeline components should be conducted, PN-EN 1254-4 (Copper and copper alloys: installation fasteners. Part 4, Threaded fas-teners/Polish Committee for Standardization Warsaw, Poland, 2021) [16] and PN-EN 1254-20 (Copper and copper alloys: installation fasteners. Part 20: Definitions, thread dimensions, test methods, reference data and supporting information Warsaw, Poland, 2021) [17]. These European standards define test methods, including leak tightness under internal hydrostatic and pneumatic pressure, pull-out resistance, and integrity of fitting bodies. Hydraulic fitting tests are classified as destructive or non-destructive. To verify the quality of a hydraulic fitting, non-destructive tests are performed. Leakage testing can be performed in two ways: one method involves submerging the system in water and introducing compressed air [18,19]. Hydraulic fitting tests are classified as destructive or non-destructive. To verify the quality of a hydraulic fitting, non-destructive tests are performed. Leakage testing can be performed in two ways: one method involves submerging the system in water and introducing compressed air; the other involves constructing a hydraulic system and pressurizing it with water to a specified pressure. In both methods, the connections should be observed to detect any leaks. In the compressed air method, air bubbles are sought as an indicator of leakage. The selection of test pressure, allowable leak rate, test volume, and test time is critical and must be carefully defined in accordance with application requirements.

Research into additive manufacturing of hydraulic components reveals a duality: significant technological potential coupled with significant practical limitations. Klimek et al. [20] showed that SLM (Selective Laser Melting)-manufactured hydraulic valve components in their as-built state exhibit leaks of 3900 cm^3^/min, exceeding the ISO 6403 (Hydraulic fluid power—Valves controlling flow and pressure—Test methods, Geneva Switzerland, 1988) [21] standard (limit 1 cm^3^/min) by nearly four orders of magnitude. This anomaly is primarily due to excessive surface roughness—the Ra parameter reaches values of up to 33.92 µm compared to the required Ra < 0.4 µm for hydraulic applications, which is almost nine times higher than the requirements (4.01–33.92 µm) [20]. The observations made by Budzik et al. regarding the accuracy of threaded elements in 3D printing confirm the significant dependence of quality parameters on printing technology, layer orientation, and material selection. A comparative analysis of FDM (ABS-M30, PLA, PETG) and PolyJet (RGD 720) systems showed that although dimensional tolerances (±0.25–0.50 mm) were achieved in accordance with ISO 965-3 (ISO general purpose metric screw threads—Tolerances—Part 3: Limit deviations for screw threads, Geneva Switzerland, 1988) [22], the surface roughness parameters (Sa: 4.92–6.48 µm, Sz: 26.70–44.10 µm, where the minimum corresponds to ABS-M30 and the maximum to RGD 720) significantly exceed the requirements for hydraulic fittings. Paradoxically, RGD 720 material, using a minimum layer thickness of 0.016 mm, exhibits optimal visual quality of the thread surface while simultaneously maximizing the roughness parameter, illustrating the fundamental difficulties in transferring 3D printing precision to the functionality of hydraulic systems [23].

Klimek et al. (2022) demonstrated in their research that the morphology of porosity and wear characteristics of SLM-printed components depend on process parameters and heat treatment variants [24]. Grzelak et al. demonstrate that modifying structural properties by optimizing SLM parameters and surface treatment can significantly improve component functionality [25]. This research shows that, although direct printing of threaded components is technically feasible, practical application requires advanced post-processing (e.g., precision threading and threaded inserts) to achieve the necessary functional strength.

Three-dimensional printing methods are increasingly used to manufacture robotic components, including hydraulic elements [26]. The work of Kostuchenko et al. showed that the main problems with printed parts are their tightness and surface roughness after printing; hence, the aim of the research described in this work was to investigate the feasibility of producing hydraulic fittings using 3D printing [27]. Moreover, manufacturing hydraulic fittings via conventional machining generates substantial waste. To assess the feasibility of 3D printing pneumatic fittings, non-destructive leakage tests were conducted. Fittings were made using two 3D printing methods—SLA and FDM. For the FDM method, PETG material was used as it has good properties for working in environments with flowing or standing water. This material was chosen for its superior mechanical properties compared to PET [11]. For the SLA method, a UV-sensitive photopolymer resin was selected. This resin was chosen for its low shrinkage rate, excellent mechanical properties, and minimal surface defects after curing, all of which are critical for producing functional hydraulic fittings with tight thread tolerances. The hydraulic fitting leakage test was conducted using a test setup similar to that used in control stations at large factories, in accordance with applicable technical standards. During the experiment, pressure testing was conducted in accordance with industry standards to evaluate the sealing performance of 3D-printed fittings compared to conventionally manufactured brass fittings.

## 2. Design and Fabrication of Connectors

**Figure 1:**
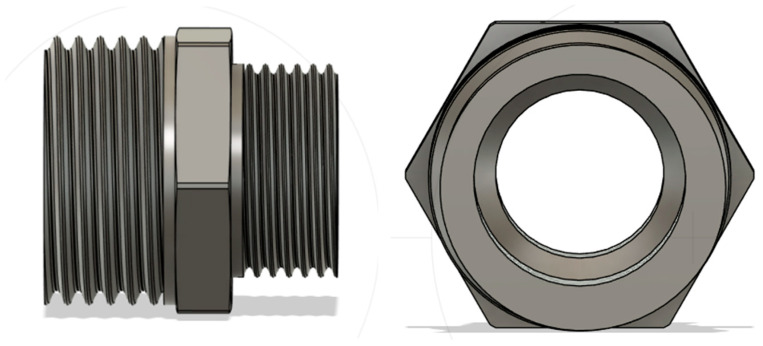
CAD model of the hydraulic connectors.

The CAD model of the tested hydraulic connectors was created in SolidWorks 2022, a program provided by Dassault Systèmes (Vélizy-Villacoublay, France) (Figure 1). The part was designed based on the existing Ferro F11Z reducer nipple as a general reducer-nipple pattern. The model includes 1/2 in and 3/8 in threads, which are standard for water fittings used to connect various household appliances. The design was created by first making a 2D sketch and adding the necessary dimensions. Subsequently, a 3D model was generated via rotational extrusion, and a hexagon was added to the ring to enable tightening with a wrench in the test system.

For the SLA printing process, a UV-sensitive photopolymer resin with a 405 nm wavelength was used. The use of this resin aimed to achieve minimal material shrinkage, high molded part strength, and an appropriately short curing time. The mechanical properties provided by the manufacturer are presented in Table 1.

The material used for FDM printing is PETG (polyethylene terephthalate glycol), a modification of traditional PET (polyethylene terephthalate). This material becomes more flexible and “the tensile strength chandes in the range of 114% to of 43.7%” [11,29]. These properties make the material impact-resistant. PETG has a low moisture absorption rate, which contributes to dimensional stability during use. Additionally, the material is UV-resistant, allowing printed parts to be used outdoors and be exposed to UV light. Due to its properties, PETG is well-suited for 3D printing, particularly for creating prototypes or functional parts. This material is also gaining popularity due to its recyclability. Studies have shown that PETG maintains mechanical stability through multiple recycling cycles, with only minor degradation after six rounds of reprocessing. The mechanical properties of resin provided by the manufacturer are presented in Table 2. The mechanical properties of the PETG filament provided by the manufacturer are presented in Table 3. Table 4 shows the 3D printing parameters set for the PETG filament.

The choice of PETG for the FDM process and UV-sensitive photopolymer resin for SLA is based on both the availability of materials and their practicality for use in laboratory research. PETG is among the most commonly used polymers in FDM printing, owing to its low filament cost and satisfactory mechanical properties (Table 3), making it accessible to academic researchers and small prototyping companies.

Similarly, UV-sensitive photopolymer resin with a 405 nm wavelength is the standard material for commercial SLA printers (FormLabs, Somerville, MA, USA; Anycubic, Shenzhen, China; Elegoo, Shenzhen, China). Due to their popularity, low purchase cost, and favorable properties (Table 1), these resins, together with PETG, are an adequate choice to meet the study’s objective of assessing whether commercially available, widely accessible additive manufacturing technologies can produce fittings with hydraulic tightness parameters.

These printing parameters were selected based on manufacturer recommendations and previous research on PETG processing to optimize layer adhesion and dimensional accuracy.

**Figure 2:**
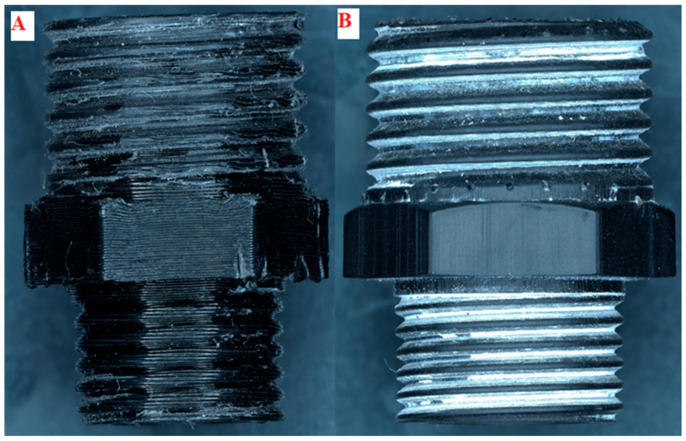
View of printed connectors at 20× zoom. (**A**) FDM sample; (**B**) SLA sample.

The printed hydraulic connectors are shown in Figure 2. The photos were taken at 20x magnification.

The material layers are shown in Figure 2, and their relative deformation is indicated.

It is also notable that certain areas exhibit protruding material layers, resulting in uneven printing and necessitating further processing to improve the thread. Thread processing involved turning down the external supports and rethreading. This post-processing technique is commonly required for FDM-printed threaded features to achieve functional thread engagement. Figure 2B shows excellent print quality, with no discernible material layer irregularities.

## 3. Development of Investigation Methodology

The study was conducted in accordance with the standards PN-EN 1254-4 and PN-EN 1254-20, which define leak tightness testing protocols for hydraulic joints. These standards specify pressure ranges below 10 bar for heating, water supply, and cooling applications. However, the relevance of our test pressure range (0.1–1.0 bar) extends beyond these conventional domains. The emerging sector of micro-hydraulics—including soft robotics and medical hydraulic devices—operates at 5–50 bar, making our selected range directly representative of realistic use cases in this specialized domain. Consequently, this study establishes baseline performance benchmarks for micro-hydraulic applications rather than conventional industrial hydraulic systems, which typically operate at 200–400 bar. The parameters for conducting the test are as follows:

- The pressure should be 1.5 times the maximum working pressure in the system (1.5 × 1.0 MPa);
- Time under pressure: 15 min;
- Water or air temperature: 23 °C +/− 5 °C.

**Figure 3:**
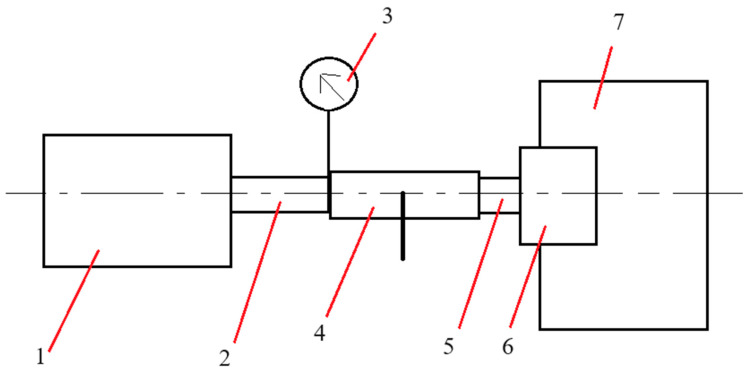
The schema of the experimental setup. The setup consists of 1—a compressor supplying pressurized air; 2—a flexible pipe; 3—a proportional pressure-reducing valve (Metalwork Regtronic); 4—a ball valve; 5—the connector being tested; 6—a blind nipple; and 7—a protective cover for the blind nipple.

These test parameters align with European Standard PN-EN 1254-20, which specifies leak tightness testing procedures for copper and copper alloy plumbing fittings under both hydrostatic and pneumatic pressure. Using 1.5 times the working pressure is a standard safety factor for pressure vessel testing. The working medium in the test system was air. The measurement system was placed in the tank, which was filled with water. The schematic of the experimental setup is shown in Figure 3.

**Figure 4:**
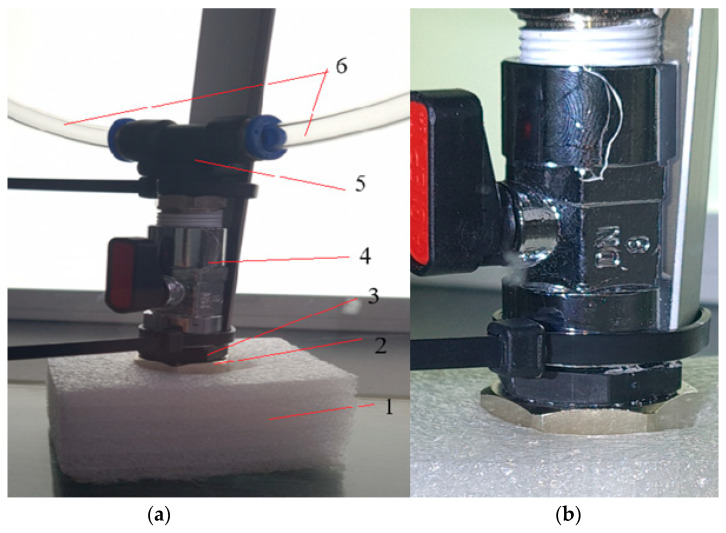
Photos of the measurement system: (**a**) 1—the protective cover for the blind nipple; 2—the blind nipple; 3—the tested connector; 4—the ball valve; 5—the quick pneumatic connector; 6—flexible pipes. (**b**) A close-up view of the tested connector.

During the experiment, the test stand was powered by an air pump with a pressure tank. The pressure in the test stand was set using a proportional pressure-reducing valve from Metalwork Regtronic (Concesio, Italy). The air pressure was set to 0.3 bar, with an accuracy of 0.5%. Using flexible pipes, the Metalwork Regtronic was connected to the ball valve. The ball valve is used to make easy changes to the investigated connectors and to protect the system against uncontrolled air leaks. The hydraulic connector, on the one side, was screwed into the valve. On the second side, it was closed with a blind nipple. For safety reasons, the blind nipple was placed in the protective cover, made of EPP foam. A photo of the measurement system is shown in Figure 4.

All threaded connections were sealed using PTFE (polytetrafluoroethylene) tape (Figure 4). PTFE tape is a standard sealing material for threaded pipe connections due to its chemical inertness, low friction coefficient, and ability to fill micro-gaps at thread interfaces. Before conducting experimental investigations, we verified the tightness of the connections. For this verification, we installed a brass base hydraulic connector. Bronze fittings are suitable benchmarks because they combine high mechanical performance with robust corrosion resistance under demanding service conditions [30].

## 4. Results of Experimental Investigations

### 4.1. Verification of the Quality of Manufactured Hydraulic Connectors

Before the tests began, we verified the dimensions and quality of the threads. The dimensions of the hydraulic connectors were measured using calipers.

Additionally, the shrinkage (relative change in dimension) of the elements after printing was determined in relation to the nominal CAD dimensions. For selected threaded-connection dimensions, linear shrinkage was calculated using Formula (1). Measurements were performed after conditioning the samples at room temperature to limit the effect of material relaxation. The nominal dimensions of the base connector and the dimensions of the connector produced by the 3D printing method with its shrinkage are presented in Table 5.

(1) $$S=\frac{L_{CAD}-L_{M}}{L_{CAD}}*100$$

where L_CAD_ [mm]—nominal dimensions; L_M-SLA_ [mm]—dimensions after SLA printing; S_SLA_ [%]—shrinkage for SLA printing; L_M-FDM_ [mm]—dimensions after FDM printing; and S_FDM_ [%]—shrinkage for FDM printing.

**Figure 5:**
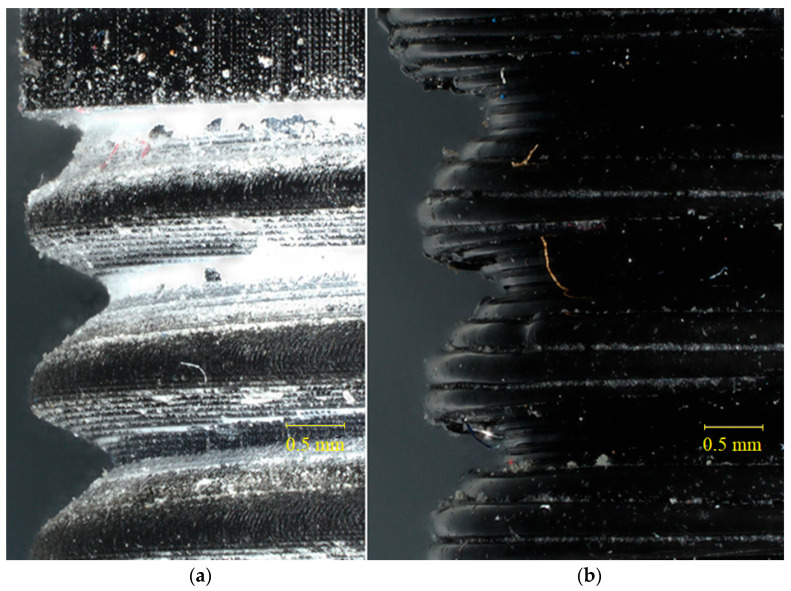
Photos comparing two printing methods for a 3/8 in thread. (**a**) SLA 3D printing method; (**b**) FDM 3D printing method.

Major internal diameter (upper reducer section) means the diameter of the reducer’s orifice through which the fluid flows, located on the reducer’s threaded side, 1/2 inch. Minor internal diameter (upper reducer section) means the diameter of the reducer’s orifice through which the fluid flows, located on the reducer’s threaded side, 3/8 inch. The nominal dimensions of the connectors presented in Table 4 are average values calculated from 5 samples. The shrinkage after printing for SLA is around 1% for resin prints. This is less than the manufacturer’s specified amount, indicating that the print was executed correctly. For FDM prints, PETG shrinkage is around 2%, which may be due to the material’s cooling method and the thickness of layers applied on top of each other. Photos comparing two printing methods for a 3/8-inch thread are shown in Figure 5.

**Figure 6:**
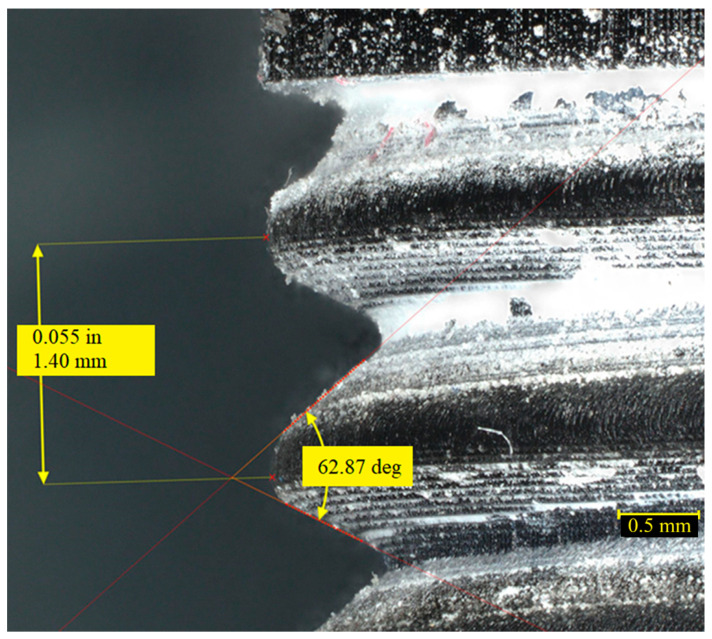
Thread outline of a 3/8″ SLA-printed connector with described angle (62.87°) and pitch (1.4 mm).

The magnification in the photos presented in Figure 5 is equal to x50. A higher-quality thread was achieved for the hydraulic fitting produced using the SLA method (Figure 5a,b). In the case of FDM printing, material layers are visible and they create voids in the material where the thread occurs (Figure 5b). These interlayer voids are characteristic of FDM technology and represent the primary mechanism of fluid leakage in pressure applications. Layer-by-layer deposition produces anisotropic mechanical properties and microchannels that permit fluid permeation even when the surface appears smooth. In Figure 6, the thread outline of a 3/8″ SLA-printed connector with the described angle and pitch is shown.

**Figure 7:**
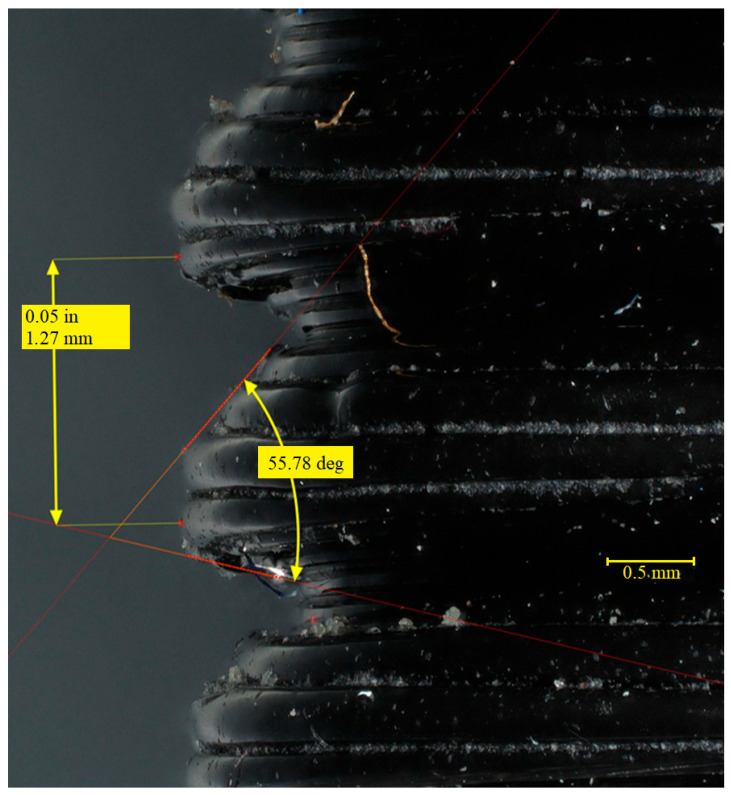
Thread outline of a 3/8″ FDM-printed connector with described angle (57.78°) and pitch (1.27 mm).

The microscope images shown in Figure 6 and Figure 7 illustrate how the height of the material layers affects the shape, angle, and pitch of the thread. The magnification in the photos presented in Figure 6 and Figure 7 is equal to x80. The 3/8-inch thread section visible in Figure 6 has a much larger angle than the 1/2-inch threads, which may be due to a lack of supports or machine vibrations. This angle is not symmetrical, with greater deviation caused by insufficient material curing. The pitch matches the nominal values for this type of thread. The thread outline of a 3/8″ FDM-printed connector with described angle and pitch is shown in Figure 7.

The irregularity in the thread formation may be due to the slow cooling of the deposited material, as suggested by the appearance of the thread in the upper sections (Figure 7). The reduced amount of material on the opposite side is because the print was made from bottom to top, causing the material to flow downward. For the microscope examination, the view of the fitting was rotated by 180°. The resulting thread angle is very close to the nominal angle. However, the pitch deviates from the nominal pitch, possibly due to difficulties in identifying two identical measurement points on the thread. These dimensional deviations are typical of FDM-printed threads and highlight the need for either design compensation or post-processing operations, such as tapping, to achieve functional thread engagement.

### 4.2. Results of Leak Tightness Investigations of Manufactured Hydraulic Connectors

**Figure 8:**
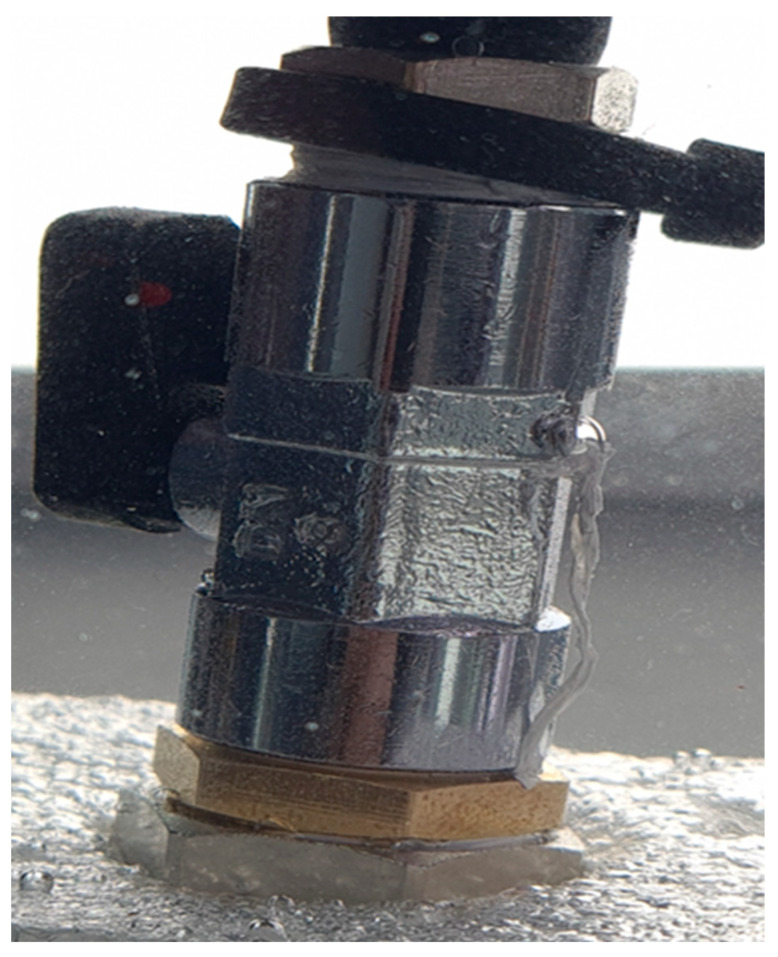
Photo showing the system operating under load with the brass connector.

To verify the quality of the hydraulic connections and the measurement system, leakage tests were first conducted on a brass hydraulic connector. The leak test was performed on both 3D-printed connectors and the available brass version of the component at pressures ranging from 0.1 to 1 bar, with and without 10 layers of PTFE tape. The hydraulic connectors were tightened using 21 mm and 24 mm open-end wrenches. Assembly torque was not controlled and is acknowledged as a limitation. The photo of the measurement system with the mounted brass hydraulic connector is shown in Figure 8.

During the leak tightness test of the hydraulic brass joint, no air bubbles were observed for the 10-layer PTFE tape case. The test results for different pressure values are presented in Table 6. The tests were performed three times for each connector to assess tightness precisely. Each time, it was resealed only with the same number of tape layers.

**Figure 9:**
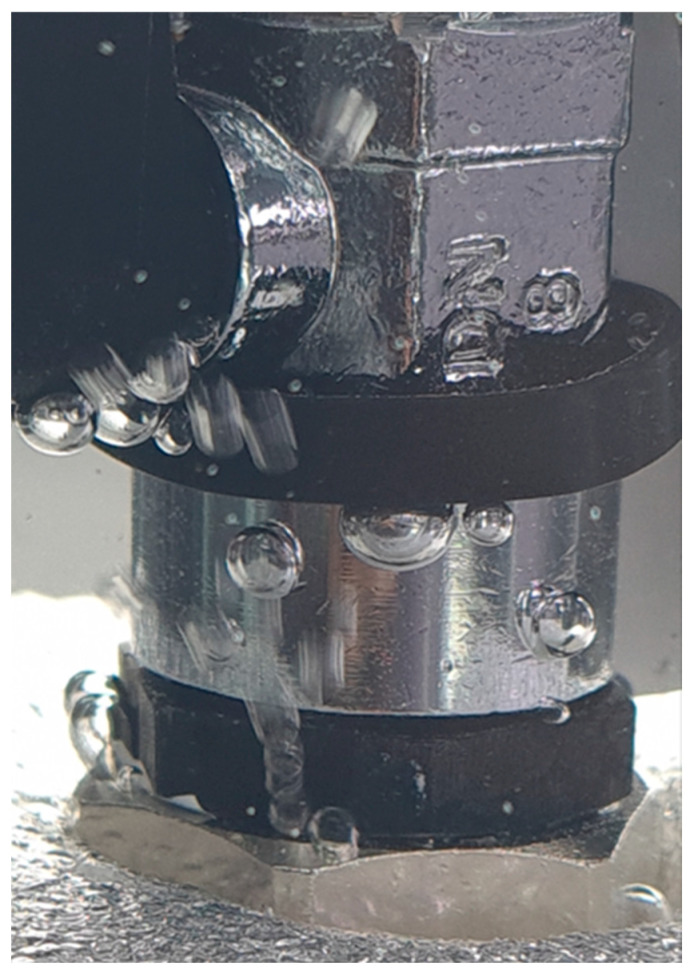
Test setup for SLA-printed fittings.

In the next step, leakage tests were conducted on a hydraulic connector manufactured via SLA 3D printing. The photo of the measurement system with the mounted hydraulic connector, manufactured via the SLA 3D printing method, is shown in Figure 9.

After placing the test system in the test vessel and initiating the experimental investigation, the system was found to be leaking in all cases analyzed. The tests were performed three times, changing the hydraulic connector each time. The test results are presented in Table 7.

**Figure 10:**
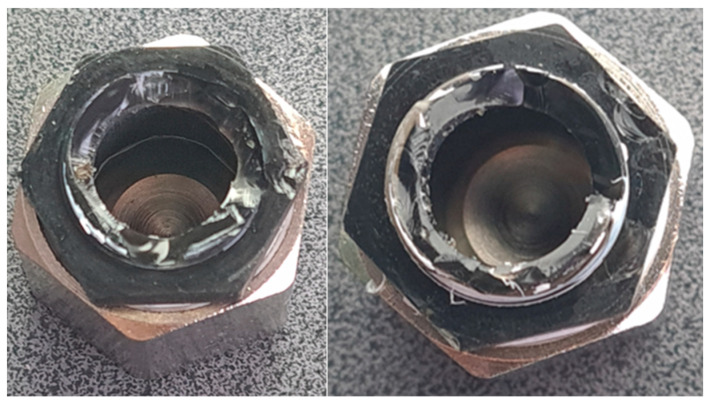
A damaged hydraulic connector made using the SLA 3D printing method.

While tightening the test assembly with wrenches, the assembly was damaged at the 3/8-inch thread location, as shown in Figure 10. This failure mode indicates insufficient mechanical strength of the photopolymer resin under the compressive and shear stresses generated during tightening. The tensile strength of the resin (36–45 MPa) is significantly lower than that of brass (up to 500 MPa), making it susceptible to thread stripping and structural failure under typical assembly torques.

**Figure 11:**
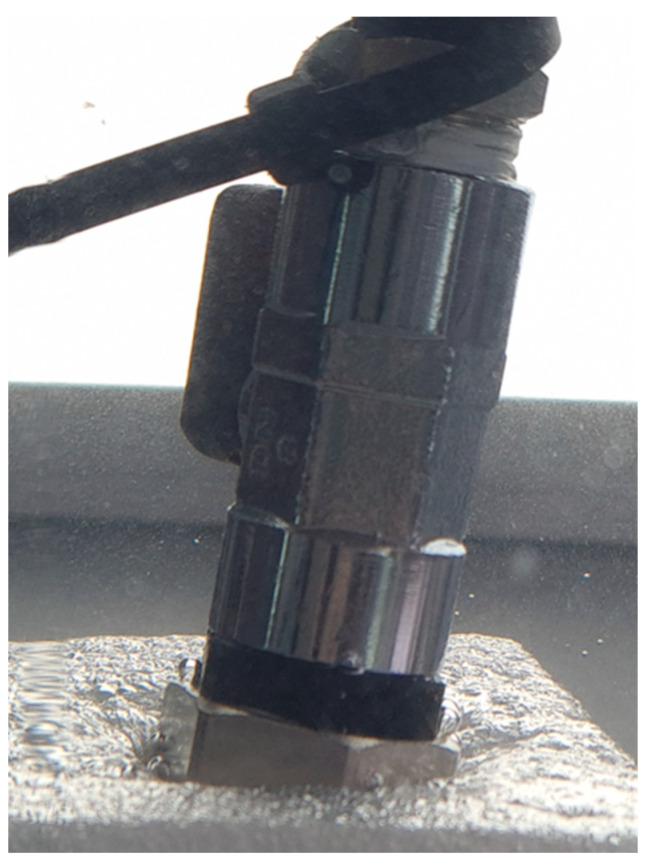
Sealed assembly with micro-leakage caused by hand tightening for FDM-printed fitting.

The following tightness tests were performed on FDM-printed connectors. The photo of the measurement system with the mounted hydraulic connector, manufactured via FDM 3D printing, is shown in Figure 11.

Similar to the previous fittings manufactured using the SLA printing method, the system was not leak-tight, is shown in Table 8.

**Figure 12:**
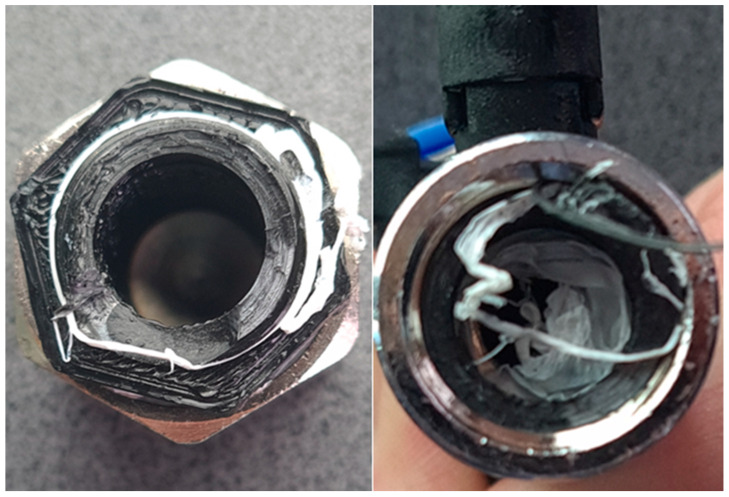
A damaged hydraulic connector made using the FDM 3D printing method.

Figure 12 shows how the assembly was damaged, though not in the exact location as the resin fittings. In this case, the layers delaminated and failed through interlayer separation. This failure mechanism is characteristic of FDM parts subjected to tensile or shear loading perpendicular to the layer orientation. The layer-by-layer construction creates planes of weakness in which adhesion between successive layers is weaker than the bulk material’s cohesive strength.

## 5. Conclusions

The leak testing studies evaluated and compared a reference brass fitting with 3D-printed fittings. The test setup was designed to assess assembly feasibility under the most demanding loading conditions within the system. None of the fittings achieved leak tightness without PTFE tape as a sealant. For the reference brass fitting, tightening achieved system sealing without causing damage. For the 3D-printed fittings, excessive tightening caused severe damage, rendering them unsuitable for further testing.

Quality control of the finished element after printing is essential for verifying dimensional geometric accuracy that conforms to the design specifications. Dimensional inspection enables assessment of the extent of shrinkage and whether it falls within tolerance. Quality control helps eliminate errors in the 3D printing process, including manufacturing inaccuracies. Proper temperature settings are particularly evident at the bottom of the part where it contacts the build platform. Careful dimensional verification allows assessment of whether a given hydraulic fitting can be adequately sealed, or whether dimensional modifications in the design are necessary and reprinting is required.

The experimental results demonstrate several key findings:

1. Dimensional accuracy: Both FDM and SLA methods achieved acceptable dimensional tolerances, with shrinkage of approximately 1% for SLA and 2% for FDM. These values are consistent with published tolerance data for these technologies.

2. Thread quality: SLA-printed threads exhibited superior surface quality and dimensional accuracy compared to FDM threads, which showed visible layer separation and porosity. However, neither method achieved thread quality sufficient for reliable sealing without post-processing.

3. Leak performance: Both 3D-printed fitting types failed to achieve leak tightness comparable to the brass reference. Micro-leakage was observed even at low pressures (0.5 bar) with PTFE sealing.

4. Mechanical strength: Both SLA and FDM fittings exhibited insufficient mechanical strength under typical assembly torques, with structural failure occurring during tightening. The failure mechanisms differed—SLA parts failed through brittle fracture at the thread root, while FDM parts failed through interlayer delamination.

These findings indicate that current polymer-based additive manufacturing technologies face significant challenges for hydraulic fitting applications. The inherent porosity of FDM parts and the lower mechanical strength of both FDM and SLA materials compared to metal fittings limit their suitability for pressure-tight fluid connections.

While 3D printing offers potential advantages in design flexibility and waste reduction for hydraulic fittings, significant technological development is required before polymer AM parts can match the performance and reliability of conventional metal fittings in pressure applications. It is also necessary to introduce changes to the fittings’ geometry during design to reduce the stresses at the junction between the threaded section and the area designed for the wrench. To verify the feasibility of manufacturing hydraulic fittings using additive manufacturing and to ensure their tightness, the fittings presented in this study were printed with a layer height of 0.16 mm. In subsequent investigations into the tightness of hydraulic fittings, we will focus on optimizing the fitting geometry, i.e., modifying the layer height and the fitting shape (notch mitigation) to increase strength during tightening. Furthermore, studies will be conducted to select appropriate fitting dimensions (accounting for layer height) to minimize, as accurately as possible, the effect of material shrinkage on the tightness of the connection.

## Figures and Tables

**Table 1:** Resin specifications provided by the manufacturer [28].

Parameter	Symbol	Value
Density	ρ	1.05–1.25 g/cm^3^
Viscosity	η	150–200 cP (mPa*s)
Surface hardness	-	84 HS
Tensile strength	σ_t_	36–45 MPa
Tensile elongation	ε_b_	11–20%
Shrinkage	α_s_	3.72–4.24%
Flexural strength	σ_f_	50–70 MPa
Flexural modulus	E_f_	1200–1600 MPa
Wavelength	λ_max_	355–410 nm

**Table 2:** Mechanical properties of resin provided by the manufacturer.

Parameter	Symbol	Value	
		Before Hardening	After 3 min of Hardening
Viscosity	η	280 cps/25	-
Hardness	-	-	82 D
Flexural strength	σ_f_	34 MPa	38 MPa
Tensile elongation	ε_b_	11%	8%
Flexural modulus	E_f_	1055 MPa	1149 MPa
Tensile strength	σ_t_	38 MPa	49 MPa

**Table 3:** Mechanical and thermal properties of PETG filament.

Parameter	Symbol	PETG Filament Properties
Tensile strength	σ_t_	25–45 MPa
Young’s modulus	E	2.0–2.3 GPa
Glass transition temperature	T_g_	80–85 °C
Tensile elongation	ε_b_	100–190%
Density	ρ	1.15–1.20 g/cm^3^
Heat deflection temperature	T_HDT_	68 °C
Odor	-	Odorless

**Table 4:** Printing parameters.

Parameter	Setting	Value
Layer Height	Medium	0.16 mm
Speed	Medium	50 mm/s
Model Structure	Normal	
Support Printing	Normal	
Printing Temperature		235 °C
Bed Temperature		60 °C
Cooling Fan Speed		50%
Material Flow		100%
Model Infill		95%
Wall Thickness		1.2 mm

**Table 5:** The nominal dimensions of the base connector and the dimensions of the connector produced by the 3D printing method with its shrinkage.

Parameter	Nominal Dimensions L_CAD_ [mm]	Dimensions After SLA Printout L_M-SLA_ [mm]	Shrinkage SLA S_SLA_ [%]	Dimensions After FDM Printout L_M-FDM_ [mm]	Shrinkage FDM [%]
External dimension on thread diameter 1/2 inch	20.8	20.6	0.96	20.5	1.44
External dimension on thread diameter 3/8 inch	16.6	16.4	1.2	16.2	2.41
Connector height	24.5	24.4	0.41	24.4	0.41
External thread height 1/2 inch	11	10.9	0.91	10.9	0.91
External thread height 3/8 inch	8.9	8.8	1.12	8.5	4.49
Dimension with a socket wrench	21	20.9	0.48	20.8	0.95
Major internal diameter (upper reducer section)	14.6	14.5	0.68	14.3	2.05
Minor internal diameter (lower nipple section)	11.6	11.5	0.86	11.3	2.59

**Table 6:** Brass hydraulic connector tightness test results.

Pressure [bar]	Sealing Teflon Tape	Obtaining Tightness	Occurrence of Sample Damage
0.1	No	No	No
0.1	Yes	Yes	No
0.5	Yes	Yes	No
1.0	Yes	Yes	No

**Table 7:** Hydraulic connector manufactured via SLA 3D printing: tightness investigation results.

Pressure [bar]	Sealing Teflon Tape	Obtaining Tightness	Occurrence of Sample Damage
0.1	No	No	No
0.1	Yes	No	No
0.5	Yes	No (micro leak)	No
1.0	Yes	No	Yes

**Table 8:** Hydraulic connector manufactured via FDM 3D printing: tightness investigation results.

Pressure [bar]	Sealing Teflon Tape	Obtaining Tightness	Occurrence of Sample Damage
0.1	No	No	No
0.1	Yes	Yes	No
0.5	Yes	Yes	No
1.0	Yes	No (micro leak)	Yes

## Data Availability

The original contributions presented in the study are included in the article, further inquiries can be directed to the corresponding author.
